# ZEB1 transcriptionally regulated carbonic anhydrase 9 mediates the chemoresistance of tongue cancer via maintaining intracellular pH

**DOI:** 10.1186/s12943-015-0357-6

**Published:** 2015-04-15

**Authors:** Guopei Zheng, Cong Peng, Xiaoting Jia, Yixue Gu, Zhijie Zhang, Yingen Deng, Chengkun Wang, Nan Li, Jiang Yin, Xiaorong Liu, Minying Lu, Hailin Tang, Zhimin He

**Affiliations:** Cancer Hospital and Cancer Research Institute of Guangzhou Medical University, Hengzhigang Road 78#, Guangzhou, 510095 Guangdong China; Department of Breast Oncology, Sun Yat-Sen University Cancer Center, State Key Laboratory of Oncology in South China, Collaborative Innovation Center for Cancer Medicine, East Dongfeng Road 651#, Guangzhou, 510060 Guangdong China

**Keywords:** Carbonic anhydrase 9, ZEB1, Intracellular pH, Tongue cancer, Chemoresistance

## Abstract

**Background:**

Chemoresistance is a major obstacle in successfully treating cancers, and the mechanisms responsible for drug resistance are still far from understood. Carbonic anhydrase 9 (CA9) has been shown to be upregulated in the drug-resistant tongue cancer cell line Tca8113/PYM and to be associated with drug resistance. However, the mechanisms regulating CA9 expression and its role in drug resistance remain unclear.

**Methods:**

Bioinformatic and experimental analysis involving ChIP and luciferase reporter assays were used to validate Zinc finger E-box-binding homeobox 1 (ZEB1) as a transcriptional regulator of CA9. Gene expression and protein levels were evaluated by quantitative RT-PCR and western blotting, respectively. Sensitivity to chemotherapy was examined using the MTS assay and Hoechst staining and analysis caspase-3 activity to evaluate changes in apoptosis. Intracellular pH (pHi) was measured using fluorescent pH-indicator BCECF-AM. Protein expression in patient tissue samples was examined by immunohistochemistry and survival of tongue cancer patients from which these samples were derived was also analyzed.

**Results:**

ZEB1 bound to the promoter of *CA9* to positively regulate *CA9* expression in tongue cancer cells. Knockdown of CA9 using short interfering RNA (siRNA) abolished the chemoresistance resulting from ZEB1 overexpression in Tca8113 and SCC-25 cells, and CA9 overexpression attenuated chemosensitivity induced by ZEB1 knockdown in Tca8113/PYM cells. CA9 knockdown also prevented maintenance of pHi mediated by overexpression of ZEB1 in Tca8113 and SCC-25 cells following chemotherapy, associated with increased apoptosis and caspase-3 activation. Conversely, ectopic expression of CA9 suppressed decrease in pHi mediated by ZEB1 knockdown in Tca8113/PYM cells following chemotherapy, accompanied by decreased apoptosis and caspase-3 activation. Importantly, a positive correlation was observed between ZEB1 and CA9 protein expression in tongue cancer tissues, and expression of these proteins associated with a poor prognosis for patients.

**Conclusion:**

Our finding that tumor cells regulate pHi in response to chemotherapy provides new insights into mechanisms of drug resistance during cancer treatment. Identification of the ZEB1–CA9 signaling axis as a biomarker of poor prognosis in tongue cancer will be valuable in future development of therapeutic strategies aimed at improving treatment efficacy, especially in terms of drug resistance associated with this disease.

## Background

Squamous cell carcinoma (SCC) of the oral cavity represents the tenth most frequent solid cancer worldwide [1]. Tongue cancer is the most common type of oral cancer and frequently leads to the malfunction of mastication, speech, and deglutition [2]. As one of the standard therapeutic approaches, chemotherapy mostly based on pingyangmycin (PYM) and/or cisplatin (cDDP), plays an important role in tongue cancer treatment and brings many benefits including reducing tumor size, inhibiting distant metastasis and prolonging patient survival [3]. However, resistance to anticancer agents is a major obstacle for successful chemotherapy in tongue cancer, as it can result in more aggressive tumor behavior and worse clinical outcome [4,5]. The molecular basis of sensitivity and resistance to chemotherapy is complex, involving multiple biological processes such as drug transport, drug metabolism, apoptosis and DNA repair [6]. Although the mechanisms responsible for drug resistance in cancer have been the subject of intense studies for decades, the clinical causes of drug resistance remain poorly understood. There is, therefore, an urgent need to clarify these mechanisms and to explore alternative therapeutic strategies to overcome drug resistance in tongue cancer treatment.

We have previously shown that carbonic anhydrase IX (CA9) expression is increased in the pingyangmycin (PYM)-induced multidrug-resistant human tongue squamous cell carcinoma cell line Tca8113/PYM when compared to the parental cell line Tca8113, without significant expression difference in multidrug resistance genes such as MDR, BCRP and MRP. Functional experiments demonstrated that both carbonic anhydrase inhibitor application and knockdown of CA9 expression enhanced PYM chemosensitivity [7]. In agreement with our previous study, Michael et al. have suggested that CA9 expression in squamous cell head and neck tumors is associated with resistance to chemoradiotherapy [8]. CA9 overexpression has been identified in a number of solid tumors, including renal carcinomas, cervical squamous carcinomas, esophageal carcinomas, bladder carcinomas and non-small cell lung carcinomas. CA9 as a membrane-associated protein is a zinc metalloenzyme catalyzing the reversible conversion of CO_2_ to bicarbonate and a proton, and this has the effect of reducing local extracellular pH [9]. Under equivalent culture conditions, the extracellular pH (pHe) of the culture media of Tca8113/PYM cells is much lower than that found for the culture media of Tca8113 cells, and this can be attributed to the increased expression of CA9 in the Tca8113/PYM cell line [7]. Generally, solid cancers maintain a high intracellular pH (pHi) but a low extracellular pH (pHe) that results from hypoxia or acidosis. Adaptation of cancer cells to hypoxia and acidosis is a critical driving force in tumor progression and metastasis [10,11]. Cancer cells have developed key strategies to regulate their pHi, because a pHi variation of as little as 0.1 can disrupt multiple biological functions including ATP production, protein synthesis, cell proliferation, migration and apoptosis [12-14]. Whether cellular adaptation associated with drug resistance is due to CA9 overexpression requires further investigation. CA9 expression is strongly induced by hypoxia-inducible factor 1 (HIF-1) and is believed to be involved in cancer cell proliferation, transformation and survival, making it a potential target for cancer therapy [15,16]. However, there is no significant difference in the expression of HIF-1 between Tca8113 and Tca8113/PYM cells, implying some other factors are responsible for CA9 overexpression in Tca8113/PYM cells. In the present study, we investigated the expression patterns of, and correlation in expression between, CA9 and ZEB1 in tongue cancer cell lines and tissues. Bioinformatics analysis combined with experimental validation demonstrated that ZEB1 transcriptionally regulates CA9 expression and that ZEB1 may contribute to chemotherapy resistance via CA9-dependent modulation of pHi.

## Results

### ZEB1 transcriptionally regulates CA9 expression in tongue cancer cells

**Figure 1:**
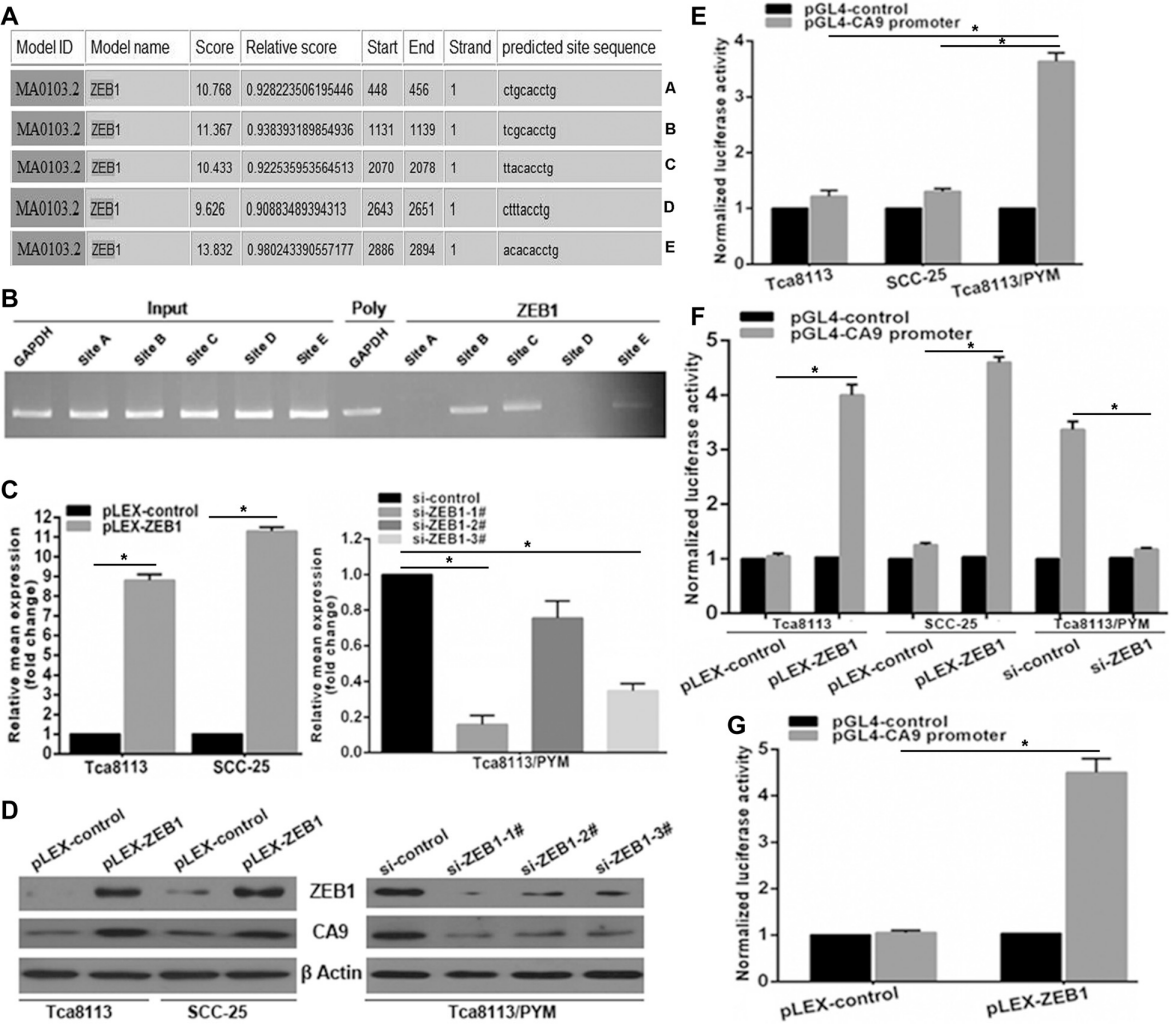
CA9 is upregulated by ZEB1 in tongue cancer cells. **(A)** A schematic representation of ZEB1 binding sites with the E-box sequence (CACCTG) in the 3kb putative *CA9* promoter. The first base of the 3kb strand is defined as ‘1’. **(B)** Chromatin immunoprecipitation assays identified ZEB1 binding sites within the putative *CA9* promoter. Primers specific for sites B, C and E yielded PCR reaction products from ZEB1–DNA immunoprecipitates. The input represents DNA directly after lysis. The PCR reaction product for immunoprecipitates obtained using the RNA Polymerase antibody represents the positive control. **(C and D)** Changes in CA9 mRNA and protein expression following the inhibition of ZEB1 in tongue cancer cell lines were evaluated by qRT-PCR and western blotting, respectively. **(E)** Luciferase activity driven by the putative *CA9* promoter was higher in Tca8113/PYM cells (which exhibit endogenous ZEB1 overexpression) than in Tca8113 and SCC-25 cells. **(F)** Reporter assays revealed changes in luciferase activity after inhibition of ZEB1 expression in tongue cancer cells. **(G)** ZEB1 promoted luciferase activity driven by the putative *CA9* promoter in HEK283T cells. * p < 0.01.

We have previously shown that both CA9 mRNA and protein expression is upregulated in the PYM-induced multidrug-resistant tongue cancer cell line Tca8113/PYM. To determine whether transcriptional regulation contributes to CA9 upregulation in Tca8113/PYM cells, we analyzed the response elements of a cohort of transcription factors located within a three kilobase region upstream of the first exon of the *CA9* gene. Using the JASPAR database (http://jaspar.binf.ku.dk) we identified five putative ZEB1 binding sites within this region, conforming to the optimal recognition sequence of ZEB1 (CACCTG) (Figure 1A). To confirm the direct association of ZEB1 with the *CA9* promoter, we performed a ChIP assay in Tca8113/PYM cells for all putative ZEB1 binding sites within the three kilobase region. ChIP results revealed that ZEB1 bound most significantly to sites B, C and E within the potential *CA9* promoter (Figure 1B). As expected, ectopic expression of ZEB1 using the pLEX-ZEB1 construct enhanced both CA9 mRNA and protein expression in Tca8113 and SCC-25 cell lines (Figure 1C and D). Conversely, CA9 mRNA and protein expression decreased following knockdown of ZEB1 in Tca8113/PYM cells using ZEB1-specific siRNAs (Figure 1C and D). To investigate further the effects of ZEB1 on CA9 expression, the putative three kilobase *CA9* promoter was cloned into a luciferase reporter vector and expression assays subsequently performed. As expected, *CA9* promoter-driven luciferase activity was much higher in Tca8113/PYM cells than in Tca8113 and SCC-25 cells (Figure 1E). In addition, ZEB1 was found to significantly enhanced luciferase activity driven by the *CA9* promoter in HEK293T cells (Figure 1G). These results demonstrate that ZEB1 can directly bind to the *CA9* promoter to transcriptionally regulate CA9 expression.

### CA9 contributes to ZEB1-mediated drug resistance in tongue cancer cells

**Figure 2:**
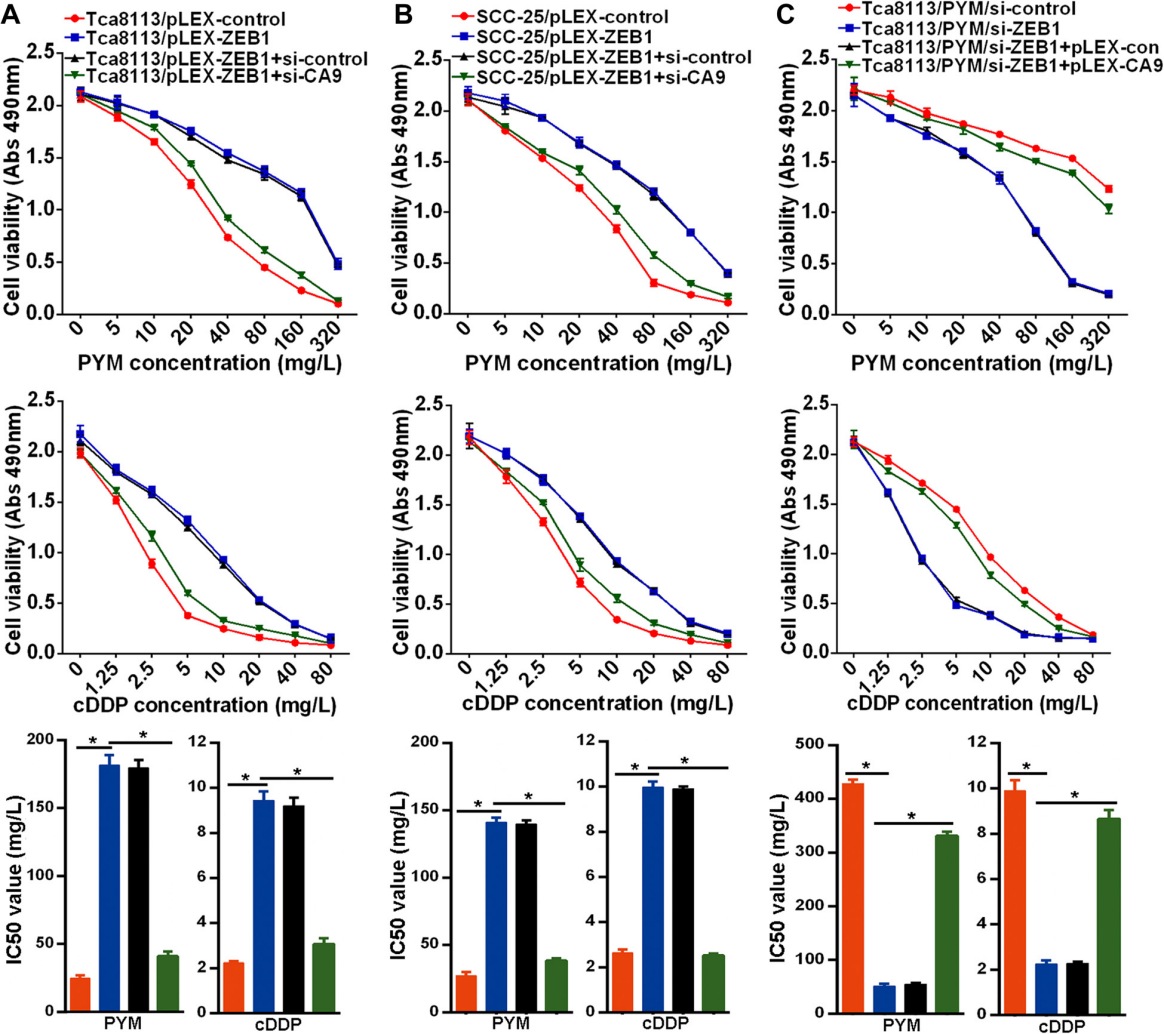
The ZEB1–CA9 axis regulates chemosensitivity in tongue cancer cells. **(A and B)** MTS cell proliferation assays showed that ZEB1 overexpression promoted resistance in Tca8113 and SSC-25 cells in response to PYM and cDDP, and that knockdown of CA9 abolished this effect. **(C)** Knockdown of ZEB1 enhanced the sensitivity of Tca8113/PYM cells to PYM and cDDP, while overexpression of CA9 attenuated this effect. * p < 0.01.

We have previously shown that CA9 is involved in drug resistance in tongue cancer cells, and have shown here that ZEB1 positively regulates CA9 expression. Next, we wished to determine whether ZEB1 modulates chemosensitivity in tongue cancer and whether it exerts its effect via regulating CA9 expression. We found that ectopic ZEB1 expression enhanced the resistance of Tca8113 and SCC-25 cells to PYM or cDDP, with the marked increase of IC50 values (Figure 2A and B). Conversely, ZEB1 knockdown increased the sensitivity of Tca8113/PYM cells to PYM and cDDP, with the significant decrease of IC50 values (Figure 2C). Moreover, CA9 knockdown markedly impaired ZEB1-mediated drug resistance to PYM or cDDP in Tca8113 and SCC-25 cells (Figure 2A and B), while overexpression of CA9 in Tca8113/PYM cells impaired the effect of ZEB1 knockdown in response to chemotherapy (Figure 2C), suggesting CA9 is responsible for ZEB1-mediated drug resistance in tongue cancer cells.

### The ZEB1–CA9 axis regulates chemotherapy-induced changes in intracellular pH

**Figure 3:**
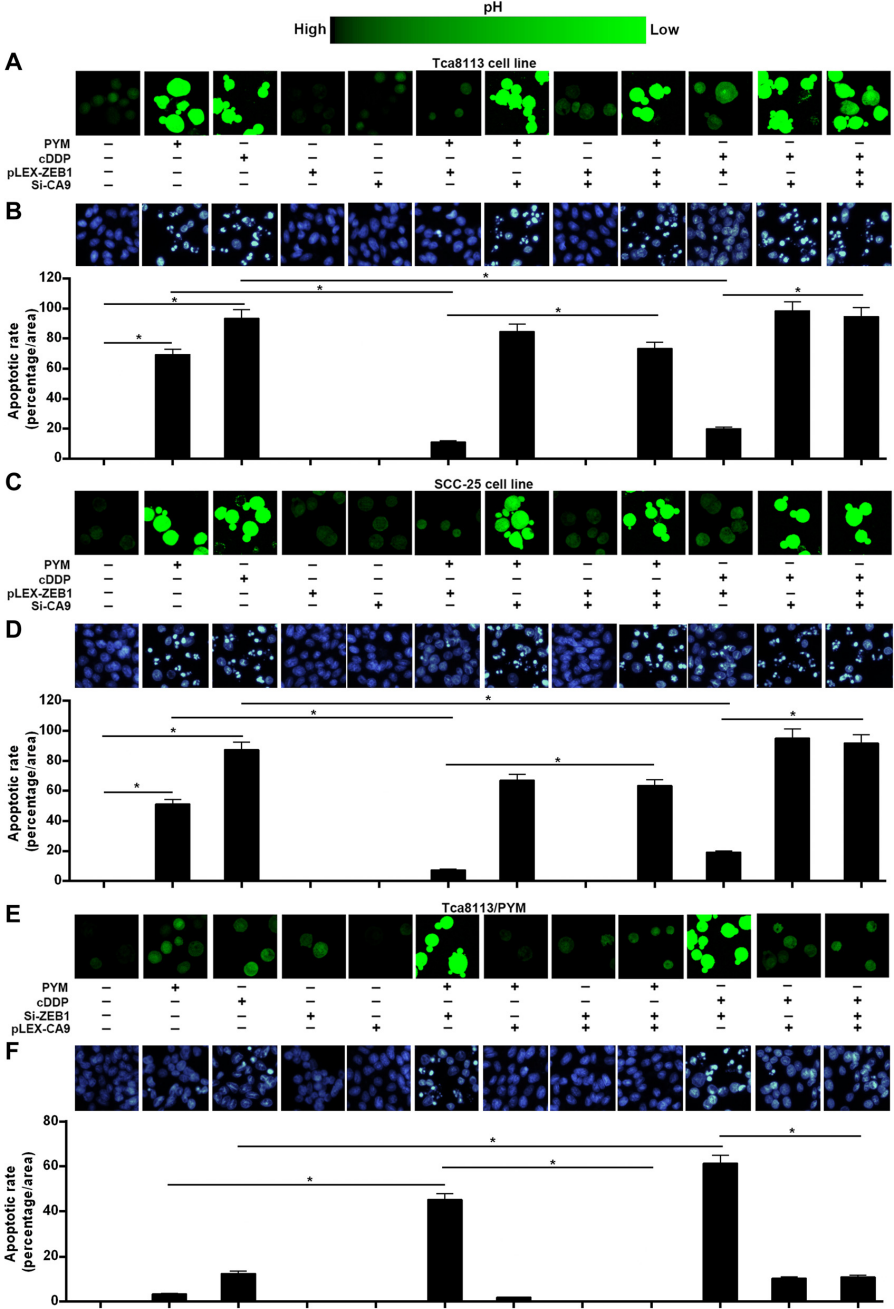
The ZEB1–CA9 axis prevents pHi decrease induced by chemotherapy in tongue cancer cells. **(A, C and E)** The change in pHi found for each given cell line in response to PYM or cDDP treatment, as determined using the BCECF-AM pH fluorescence probe in conjunction with confocal microscopy. **(B, D and F)** Changes in apoptosis in response to PYM or cDDP treatment for each given cell line, as determined by Hoechst staining evaluated by fluorescence microscopy. * p < 0.01.

Tumor cells usually have a higher pHi (which is neutral-to-alkaline) in comparison to normal cells [17]. Alterations in pHi homeostasis have been implicated in anticancer drug treatment and drug-resistant cancer cells may develop multiple mechanisms to regulate pHi more effectively in response to extracellular or intracellular stress. Here, we found that PYM (80mg/L) or cDDP (5mg/L) treatment resulted in a significant decrease in the pHi (Figure 3A and C) and an accompanying increase in apoptosis (Figure 3B and D) in Tca8113 and SCC-25 cells when compared to Tca8113/PYM cells (Figure 3E and F). Ectopic ZEB1 expression markedly impaired the PYM or cDDP-induced decrease in pHi and the associated increase in apoptosis in Tca8113 and SCC-25 cell lines (Figure 3A–D). However, knockdown of CA9 in these cells attenuated the effect of ectopic ZEB1 expression on pHi and apoptosis following PYM or cDDP treatment (Figure 3A–D). Moreover, in Tca8113/PYM cells, knockdown of ZEB1 enhanced the PYM or cDDP-dependent decrease in pHi and the associated increase in apoptosis, while overexpression of CA9 essentially abolished these effects (Figure 3E and F). These findings suggest that the ZEB1–CA9 signaling axis prevents pHi decrease and apoptosis induced by chemotherapy.

### The ZEB1–CA9 axis modulates chemotherapy-induced caspase-3 activation

**Figure 4:**
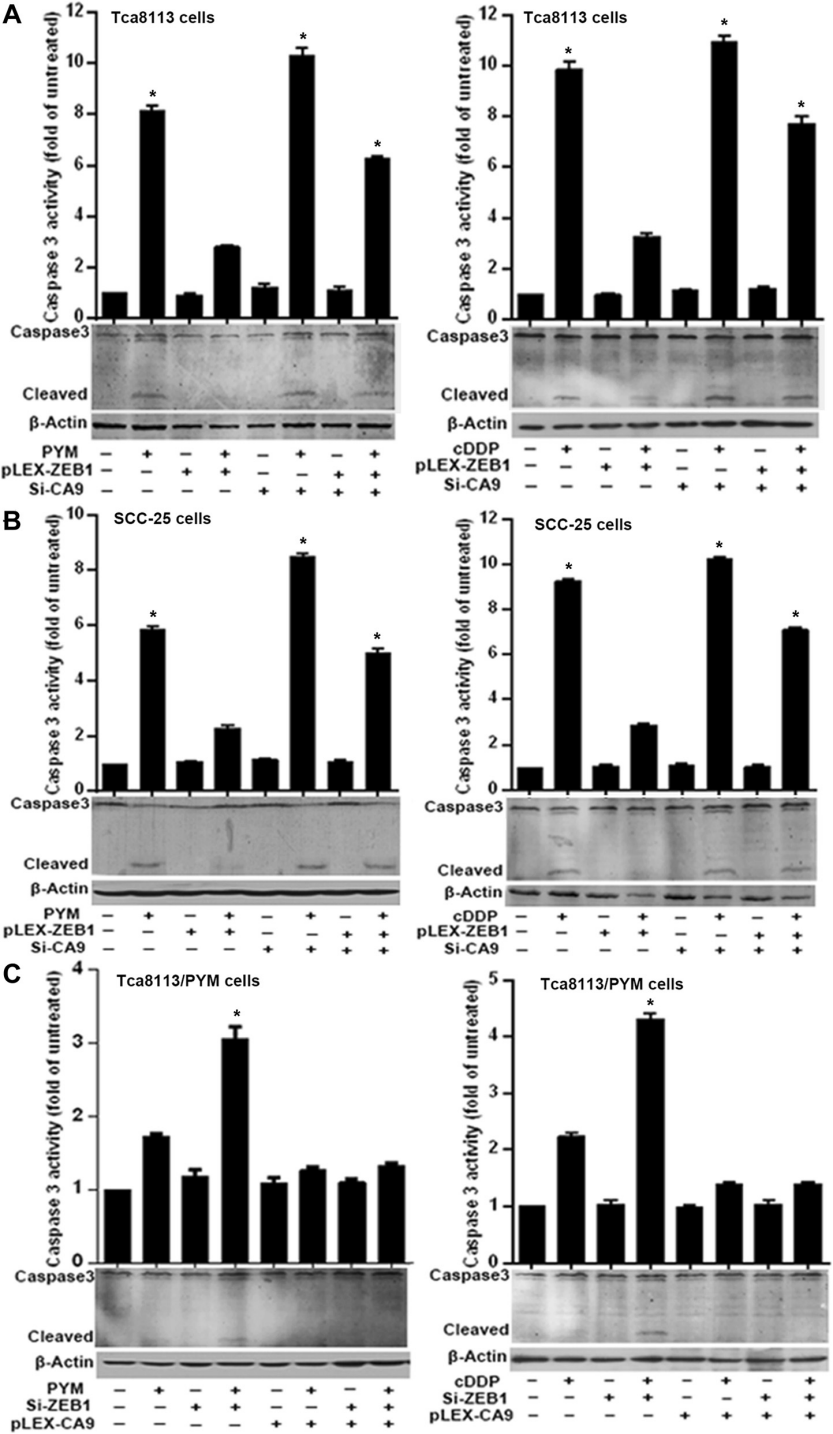
ZEB1–CA9 prevents chemotherapy-induced caspase-3 activation. **(A and B)** PYM and cDDP induced caspase-3 activation in Tca8113 and SCC-25 cell lines, respectively, as measured by reporter assays and western blotting. Overexpression of ZEB1 prevented caspase-3 activation in response to chemotherapy, while knockdown of CA9 impaired the effects of ZEB1 overexpression. **(C)** Chemotherapy had no significant effect on caspase-3 activation in Tca8113/PYM cells. Knockdown of ZEB1 enhanced caspase-3 activation induced by chemotherapy, and overexpression of CA9 attenuated this effect. *vs.* no treatment, * p < 0.01.

A decrease in pHi correlates with an increase in apoptosis and caspase-3 is a key component of the apoptotic pathway [18]. We therefore performed a caspase-3 activity assay and western blotting to detect the cleaved, active protein and therefore determine the functional state of caspase-3 in cultured cells. We found that PYM (80mg/L) or cDDP (5mg/L) treatment significantly promoted caspase-3 activation in Tca8113 and SCC-25 cells (Figure 4A and B) and this was accompanied by a decrease in pHi (Figure 3A and B). With respect to the change in pHi, we found that ectopic ZEB1 expression blocked PYM or cDDP-induced caspase-3 activation in Tca8113 and SCC-25 cells (Figure 4A and B), while the simultaneous knockdown of CA9 abolished this ZEB1-related effect (Figure 4A and B). Furthermore, in Tca8113/PYM cells (in which endogenous ZEB1 is upregulated) we found that knockdown of ZEB1 promoted caspase-3 activation in response to PYM or cDDP (Figure 4C). However, ectopic CA9 expression attenuated the effect of ZEB1 knockdown on caspase-3 activation (Figure 4C). These results suggest that CA9 is involved in the ZEB1-dependent inhibition of caspase-3 activation.

### ZEB1 and CA9 are negative prognostic markers in tongue cancer

**Figure 5:**
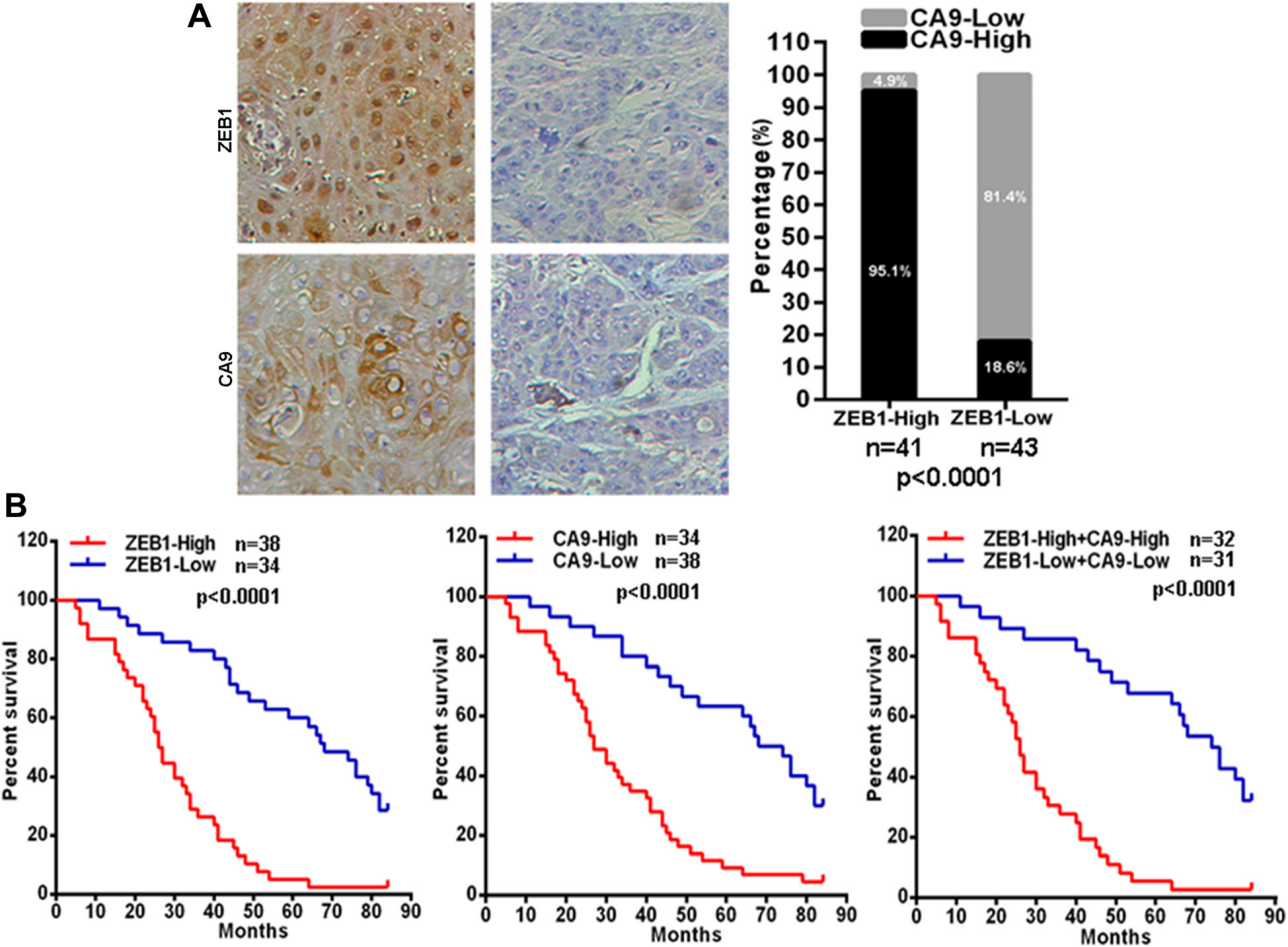
ZEB1 expression correlates with that of CA9 in tongue cancer and is associated with poor clinical prognosis. **(A)** Representative images of ZEB1 and CA9 protein expression in tongue cancer tissues. ZEB1 protein expression was positively correlated with that of CA9 (left). The level of CA9 protein expression in tongue cancer tissues that exhibit different levels of ZEB1 protein expression (right). **(B)** Kaplan–Meier analysis estimated overall survival according to the ZEB1 protein level, CA9 protein level, and both ZEB1 and CA9 protein levels.

Given the above observations in cultured cells, namely that ZEB1 transcriptionally regulates CA9 expression and that the ZEB1–CA9 axis mediates chemotherapy resistance, we next wished to assess whether there is a correlation between ZEB1 expression and CA9 expression in tongue cancer. We therefore performed immunohistochemical staining on tissue arrays containing tongue cancer tissue from 84 patients receiving chemotherapy based on PYM and/or cDDP. Notably, CA9 protein was strongly expressed in 39 of 41 tongue cancer tissues that exhibited high ZEB1 protein expression (Figure 5A). In contrast, CA9 protein was strongly expressed in only 8 of 43 tongue cancer tissues that exhibited low ZEB1 protein expression (Figure 5A). Importantly, tongue cancer patients with high ZEB1 expression have a poor prognosis for overall survival compared to patients with low ZEB1 expression (Figure 5B). In addition, high CA9 expression was also predictive of worse overall survival for tongue cancer patients (Figure 5B). Moreover, tongue cancer patients with both high ZEB1 and CA9 expression have worse overall survival compared to patients with both low ZEB1 and CA9 expression (Figure 5B). All the results denoted a potential correlation between ZEB1 and CA9 expression and their indication for the poor prognosis in human tongue cancer patients.

## Discussion

Therapeutic resistance, which often appears and which is caused by various mechanisms, is the central problem in cancer therapy [19]. This underlines the critical importance of elucidating the precise molecular mechanisms involved in the anticancer drug resistance of cancer cells for improving current treatments in the clinic. We have previously shown that overexpression of CA9 is associated with drug resistance in tongue cancer cells [7], although at that time the mechanism of CA9 upregulation was unclear. In the present study, bioinformatics analysis combined with experimental validation has revealed that ZEB1 binds to the promoter of the *CA9* gene and positively regulates its activity. ZEB1 expression was also found to promote CA9 expression in tongue cancer cells. Given the role of CA9 in drug resistance and that ZEB1 regulates the expression of CA9, we further investigated the role of ZEB1 in the drug resistance of tongue cancer cells. We found that ectopic expression of ZEB1 in tongue cancer cells significantly enhanced their resistance to chemotherapy while knockdown of ZEB1, conversely, increased sensitivity to chemotherapy. Moreover, knockdown of CA9 attenuated the effect of ectopic ZEB1 expression on the chemosensitivity of Tca8113 and SCC-25 cell lines and ectopic CA9 expression abolished the effect of ZEB1 knockdown on chemoresistant Tca8113/PYM cells.

ZEB1 has been reported to be highly expressed in epithelial cancers and its expression correlates with poor prognosis [20]. ZEB1 is a known driver of epithelial-to-mesenchymal transition (EMT), a phenotype associated with cancer cells that are typically prone to metastasis, drug resistance and poor clinical outcome, and which represent potential therapeutic targets for cancer progression. ZEB1 represses the expression of epithelial genes and certain micro RNAs (miRNAs), including the miR-183, miR-203 and miR-200 family members, which function not only as strong inducers of EMT but also as inhibitors of stem cell properties [21,22]. ZEB1 knockdown reverses the EMT phenotype, inhibits migratory ability and enhances the chemosensitivity of docetaxel-resistant human lung adenocarcinoma cells [23]. The ZEB1 pathway links glioblastoma initiation and invasion. ZEB1 expression in glioblastoma patients is predictive of shorter survival and poor temozolomide response [24]. Moreover, ZEB1 has also been reported to be closely associated with radioresistance [25,26]. We now show here that ZEB1 predominantly correlates with drug resistance, mainly via modulating CA9 expression in tongue cancer cells.

CA9 has been reported to be overexpressed in many types of solid tumors and contributes to low pHe [9], as observed in our experimental model [7]. The cancer cells of solid tumors usually developed the ability to maintain a high pHi and a low pHe to adapt and sustain normal biological process, as intracellular acidification is closely associated with apoptosis. A change of pHi/pHe ratio of 0.1-0.2 pH units can have disastrous consequences for critical biological processes including ATP synthesis, proliferation, metastasis and survival [27-29]. Nitric oxide-induced acidification drives nitric oxide-induced apoptosis in neurons [30]. Studies from Rich et al. have shown that leukemic cell lines and peripheral blood from primary patient leukemic samples exhibited a constitutively higher pHi than normal hematopoietic tissue, and that a pHi decrease enhanced apoptosis in leukemic cells [18]. Caspase activation plays a critical role during apoptosis. The efficiency of caspase activation by cytochrome C is pH-sensitive, and lower pH drives this activation [31]. With respect to CA9, Chiche et al. have shown that forced expression of CA9 contributes to extracellular acidification and to the maintenance of a more alkaline pHi [32]. The primary enzymatic function of CA9 is to catalyze the reversible hydration of carbon dioxide to bicarbonate and protons to contribute to acidification of the tumor microenvironment, acting as a “catalytic converter” for the excretion of acid from cells. Bicarbonate is shuttled into the cytoplasm to buffer pHi, while the proton remains in the extracellular space. The biological effects of CA9 help to produce and maintain an alkaline pHi favorable for tumor growth, and participate in the generation of an increasingly acidic extracellular space, facilitating tumor progression. Thus, Inhibition of CA9 interferes with removal of acid and results in a decrease in pHi, negatively influencing cell survival [33,34]. In our study here we performed a series of experiments to determine whether ZEB1–CA9 mediates drug resistance via maintaining the pHi. We found that the pHi in chemo-sensitive Tca8113 and SCC-25 cells rapidly decreased upon treatment with PYM or cDDP, whereas this was not observed for chemoresistant Tca8113/PYM cells. However, overexpression of ZEB1 prevented both the pHi decrease and caspase-3 activation induced by chemotherapy, and knockdown of CA9 abolished the effect of ZEB1 overexpression on Tca8113 and SCC-25 cells. In multidrug-resistant Tca8113/PYM cells, knockdown of ZEB1 promoted a decrease in pHi after exposure to PYM or cDDP, but this decrease was blocked following CA9 overexpression. Importantly, we found that ZEB1 protein expression strongly correlated with CA9 protein expression, and that the ZEB1–CA9 axis was a negative prognostic factor for overall survival in tongue cancer patients. Therefore, ZEB1 transcriptionally regulates CA9 expression to mediate chemoresistance in tongue cancer via modulating pHi in response to chemotherapy. Further study will now be required to elucidate the molecular mechanisms coupling ZEB1–CA9 to changes in pHi and the response to chemotherapy, and to determine whether observations made *in vitro* are reflected in the clinic. In addition, new strategies should be developed to enable the monitoring of these biochemical changes in response to chemotherapeutic agents both *in vitro* and *in vivo*.

## Conclusion

In our present study, we elucidated the mechanisms responsible for the regulation of CA9 expression, and have identified the ZEB1–CA9 axis as a mediator of resistance to chemotherapy. ZEB1–CA9 mediates chemoresistance mainly via maintaining the pHi in response to chemotherapy, providing new insights into the complex mechanisms of drug resistance. Our findings strongly suggest that the ZEB1–CA9 axis can be a prognostic biomarker for tongue cancer patients and provides a rationale for the development of anticancer intervention strategies targeting the ZEB1–CA9 axis in clinics.

## Materials and methods

### Cell culture and tissue specimens

The human tongue squamous cell carcinoma cell line Tca8113 derived from moderately differentiated human tongue squamous cell carcinoma and the stable PYM-resistant cell line Tca8113/PYM, and squamous cell carcinoma cell line SCC-25 from American Type Culture Collection, were cultured in RPMI-1640 (Gibco) containing 10% fetal bovine serum (Gibco) at 37°C in a humidified atmosphere containing 5% CO_2_. To maintain the resistance phenotype, 0.5mg/L PYM was added to the culture media of Tca8113/PYM cells. The HEK293T human embryonic kidney cell line was cultured in DMEM (Gibco) with 10% fetal bovine serum. Eighty-four tongue cancer tissue specimens were obtained from patients at the Affiliated Tumor Hospital of Guangzhou Medical University between March 2000–December 2006. Overall survival was computed from the day of surgery to the day of death or of last follow-up. The study was approved by the ethics committee of the Affiliated Tumor Hospital of Guangzhou Medical University.

### ChIP assay

The ChIP assay was performed using the EZ-CHIP™ chromatin immunoprecipitation kit (Merck Millipore). Briefly: Chromatin proteins were cross-linked to DNA by addition of formaldehyde to the culture medium to a final concentration of 1%. After a 10 min incubation at room temperature, the cells were washed and scraped off in ice-cold phosphate-buffered saline (PBS) containing Protease Inhibitor Cocktail II. Cells were pelleted and then resuspended in lysis buffer containing Protease Inhibitor Cocktail II. The resulting lysate was subjected to sonication to reduce the size of DNA to approximately 200–1000 base pairs in length. The sample was centrifuged to remove cell debris and diluted ten-fold in ChIP dilution buffer containing Protease Inhibitor Cocktail II. A 5 μl sample of the supernatant was retained as “Input” and stored at 4°C. Then 5 μg of anti-RNA Polymerase antibody (positive control, included with the kit), or anti-ZEB1 antibody (cell signal technology) were added to the chromatin solution and incubated overnight at 4°C with rotation. After antibody incubation, protein G agarose was added and the sample incubated at 4°C with rotation for an additional 2 h. The protein/DNA complexes were washed with Wash Buffers four times and eluted with ChIP Elution Buffer. Cross-links were then reversed to free DNA by the addition of 5M NaCl and incubation at 65°C for 4 h. The DNA was purified according to the manufacturer’s instructions. 50 μl of DNA was obtained for each treatment. 0.2 μl of DNA from each group was used as a template for PCR. Primers for the *CA9* promoter containing putative ZEB1 binding sites were as follows, sense: 5′-TGTTGGCCAGGCTGGTCT-3′, antisense: 5′-CCACTTGAGTGCCTCAGCCA-3′ (for site A); sense: 5′-GTAGTATACCAGTCAGGTGTC-3′, antisense: 5′-AGAGATGGGATCTCACTGTGTT-3′ (for site B); sense: 5′-CAGTCTCAGCTCACTGCAGC-3′, antisense: 5′-GTGGGTGGTTGCTTGAGTCCAG-3′ (for site C); sense: 5′-CTGACACATACACTTGCTTTTC-3′, antisense: 5′-TGGCTGAGAGGGAAAGCAGCTC-3′ (for site D); sense: 5′-CCTGCATAGTGCCAGGTGG-3′, antisense: 5′-GAGATGGAGCCAAAGTCTCACAG-3′ (for site E). Primers for the human GAPDH gene: sense, 5′-TACTAGCGGTTTTACGGGCG-3′, antisense, 5′-TCGAACAGGAGGAGCAGAGAGCGA-3′. The PCR conditions were as follows: 1 cycle of 95°C for 5 min; 32 cycles of 95°C for 20 s, 59°C for 30 s, and 72°C 30 s; and 1 cycle of 72°C for 10 min. PCR samples were resolved by electrophoresis in a 2% agarose gel and stained with ethidium bromide.

### Cells transfection

Cells were trypsinized, counted and seeded into six-well plates the day before transfection to ensure 70% cell confluency on the day of transfection. The transfection of the pLEX-ZEB1 vector, the pLEX-CA9 vector and related controls was carried out using Lipofectamine 2000 (Invitrogen) in accordance with the manufacturer’s instructions. siRNAs targeting ZEB1 or CA9 and siRNA controls were purchased from Santa Cruz Biotechnology. Transfection of siRNA (50 nM final concentration) was performed as above. Experiments were performed 48 h post-transfection.

### Real-time PCR

Total RNA was extracted using Trizol, and cDNAs subsequently synthesized from mRNA templates using the Super-Script first-strand synthesis system (Thermo Scientific). Real-time PCR was carried out according to standard protocols using an ABI 7500 with SYBR Green detection (Applied Biosystems). GAPDH was used as an internal control and the qRT-PCR was repeated three times. The primers for GAPDH were: forward primer 5′-ATTCCATGGCACCGTCAAGGCTGA-3′, reverse primer 5′-TTCTCCATGGTGGTGAAGACGCCA-3′; primers for CA9 were: forward primer 5′- GTCCAGCTGAATTCCTGCCT-3′, reverse primer 5′-CCTTCTGTGCTGCCTTCTCA-3′.

### Western blotting

Total protein was extracted from cells using RIPA buffer (Pierce) in the presence of protease inhibitors (Protease Inhibitor Cocktail, Pierce). The protein concentration of lysates was measured using a BCA Protein Assay Kit (Pierce). Equivalent amounts of protein were mixed with 5 × Lane Marker Reducing Sample Buffer (Pierce), and resolved by electrophoresis in a 10% SDS–polyacrylamide gel and then transferred onto Immobilon-P Transfer Membrane (Millipore). The membranes were blocked with 5% non-fat milk in Tris-buffered saline and then incubated with primary antibodies followed by secondary antibody. The signal was detected on the Odyssey instrument (LI-COR Bioscience). ZEB1, CA9, Caspase-3, and β-Actin antibodies were from Cell Signaling Technology, and the fluorescently labeled secondary antibodies were from LI-COR Bioscience.

### Promoter activity analysis

To determine whether ZEB1 regulates the promoter activity of CA9, a three kilobase region upstream of the first exon of *CA9* was cloned into the pGL4-reporter vector upstream of the luciferase gene. Cells were seeded in 96-well plates and co-transfected with the pGL4-reporter vector and the pRL-TK Renilla luciferase vector with or without the pLEX-ZEB1 vector using Lipofectamine 2000 (Invitrogen). After 48 h, luciferase activity was determined using a Dual-Luciferase Reporter Assay System (Promega) on the BioTek Synergy 2. Renilla luciferase activity was used as an internal control and the firefly luciferase activity was calculated as the mean +/- standard deviation after normalization relative to the Renilla luciferase activity.

### Cell proliferation assays (MTS)

The CellTiter 96 AQueous One Solution Cell Proliferation Assay kit (Promega, Madison, WI, USA) was used to determine the sensitivity of cells to PYM or cDDP. Briefly, cells were seeded in 96-well plates at a density of 4 × 10^3^ cells/well (0.2 ml/well) for 24 h before use. The culture medium was replaced with fresh medium containing PYM or cDDP at different concentrations and cells were then incubated for a further 72 h. Then, MTS (0.02 ml/well) was added. After a further 2 h incubation, the absorbance at 490 nm was recorded for each well on the BioTek Synergy 2. The absorbance represented the cell number and was used for the plotting of dose–cell number curves.

### Intracellular pH (pHi) measurement

The pHi of cells was measured using the fluorescent pH-indicator 2,7-bicarboxyethyl-5,6-carboxyfluorescein-acetoxymethylester (BCECF-AM) (Molecular Probes, Eugene, OR, USA) following the manufacturer’s protocol. BCECF is a neutral lipophilic form of bis-carboxyfluorescein which diffuses freely through the plasma membrane. Within the cell it is hydrolyzed by esterases, releasing BCECF which is retained within the cytoplasm. The fluorescence intensity of BCECF is dependent upon pH. After an incubation of 10 min, the fluorescence intensity was measured using confocal microscopy using an excitation wavelength of 535 nm.

### Hoechst staining

Following transfection cells were reseeded in fresh medium in 24-well plates. After a 24 h incubation, cells were treated with or without PYM (80mg/L) or cDDP (5mg/L) for an additional 48 h. The cells were then stained with hoechst33528, and apoptotic cells possessing significantly smaller, condensed and fragmented nuclei, were observed using a fluorescence microscope. The apoptotic cell number was determined for at least three fields-of-view for each treatment and the apoptotic rate then calculated.

### Caspase-3 activity assay

Caspase-3 activity was determined using a caspase-3 activity kit (Beyotime), in which cleavage of a colorless substrate specific for caspase-3 [Ac-DEVD-p-nitroaniline (pNA)], releases the chromophore pNA. Assays were carried out according to the manufacturer’s instructions. To evaluate the activity of caspase-3, lysates were prepared from cells after their respective treatments. Assays were performed in 96-well microtiter plates. Briefly; for each sample 10 μl of cell lysate was combined with 80 μl of reaction buffer and 10 μl of caspase-3 substrate (Ac-DEVD-pNA, 2mm). Lysates were then incubated at 37°C for 4 h and samples subsequently measured on the BioTek Synergy 2 system using an absorbance of 405 nm.

### Immunohistochemistry

A tissue array containing 84 human tongue cancer specimens was cut into 4-μm sections. The sections were dried at 62°C for 2 h and then deparaffinized in xylene and rehydrated using a series of graded alcohol washes. The tissue slides were then treated with 3% hydrogen peroxide in methanol for 15 min to quench endogenous peroxidase activity, and antigen retrieval then performed by incubation in 0.01 M sodium cirate buffer (pH 6.0) and heating using a microwave oven. After a 1 h preincubation in 10% goat serum, the specimens were incubated with primary antibody overnight at 4°C. The tissue slides were treated with a non-biotin horseradish peroxidase detection system according to the manufacturer’s instruction (DAKO). Two different pathologists evaluated the immunohistological samples.

### Statistical analysis

All statistical analyses were performed with SPSS statistical software (version 21.0; IBM). Survival curves were constructed using the Kaplan–Meier method and analyzed by the log-rank test. Significant prognostic factors identified by univariate analysis were entered into multivariate analysis using the Cox proportional hazards model. The Student’s t-test was used for comparisons and the Pearson correlation test (two-tailed) was used to investigate the correlation between ZEB1 and CA9 protein levels. Statistical significance was defined as P < 0.05.
